# Evaluation de la teneur en iode du sel de cuisine des ménages dans une région d’endémie goitreuse dans la ville de Man, ouest de la Côte d’Ivoire

**DOI:** 10.11604/pamj.2025.52.83.44530

**Published:** 2025-10-24

**Authors:** Souleymane Tandamba, Assita Yao, Anselme N'guessan, Nafi Ballo, Gnomblesson Georges Tiahou, Jacko Abodo

**Affiliations:** 1 Service d'Endocrinologie Diabétologie, Hôpital Militaire d'Abidjan, Université Félix Houphouët Boigny, Abidjan, République de Côte d'Ivoire; 2 Laboratoire de Biochimie et Biologie Moléculaire, Université Alassane Ouattara, Bouaké, République de Côte d'Ivoire

**Keywords:** Iode, sel de cuisine, teneur en iode, ménage, région d’endémie goitreuse, Iodine, cooking salt, iodine content, household, goiter-endemic area

## Abstract

Le déficit d'apport alimentaire quotidien en iode est responsable d'une série d'anomalies qualifiées de « troubles dus à la carence en iode (TDCI) » parmi lesquelles, le goitre endémique et l'hypothyroïdie. La ville de Man qui est dans une zone d'endémie goitreuse n'a fait l'objet d'aucune étude pour évaluer le niveau d'iodation du sel de cuisine dans les ménages. Evaluer la teneur en iode du sel de cuisine dans les ménages de la ville de Man, une zone d'endémie goitreuse en Côte d'Ivoire, et rechercher les facteurs influençant sa dégradation. Etude transversale descriptive sur 1 mois, portant sur le dosage du sel prélevé aléatoirement dans les ménages et dans le commerce. 160 ménages et 2 grossistes ont été inclus. La teneur en iode du sel dans les ménages était: 81% en dessous de la norme de l’OMS (<15 ppm), 85% en dessous de la norme ivoirienne (<30 ppm) et 10% de sel non iodé. La ville disposait de deux commerces dont les sels ont une teneur en iode en dessous de la norme. Les moyens de stockage tels que les flacons en plastique et en polyéthylène transparents étaient fortement associés au niveau faible d'iode dans le sel (p=0,0063). Cependant, les flacons métalliques étaient associés à un niveau d'iode normal (p=0,006). La durée de conservation n'influençait pas la dégradation de l'iode (p=0,075). Il existe une insuffisance d'iodation du sel de cuisine en dessous des normes de l'OMS, nécessitant un contrôle régulier par les autorités compétentes et la mise en place d'actions préventives. Celles-ci devront permettre de conduire de nouvelles études pour en évaluer l'efficacité.

## Abstract (en)

Insufficient daily dietary iodine intake is responsible for a range of disorders collectively referred to as iodine deficiency disorders (IDD), including endemic goiter and hypothyroidism. The city of Man, located in a goiter-endemic area, has not previously been the subject of a study evaluating the iodine content of household cooking salt. The purpose of this study was to assess iodine content in household cooking salt in the city of Man, a goiter-endemic area in Côte d'Ivoire, and to identify factors contributing to iodine degradation. We conducted a one-month descriptive cross-sectional study focused on measuring the iodine content of salt samples randomly collected from households and commercial outlets. A total of 160 households and 2 wholesale suppliers were included. The iodine content of household salt was found to be below WHO standards (<15 ppm) in 81% of cases, below the national standard (<30 ppm) in 85% of cases, and entirely non-iodized in 10% of samples. The city had two retail outlets, both of which sold salt with iodine content below the recommended standard. Storage methods using transparent plastic and polyethylene containers were strongly associated with low iodine levels in salt (p=0.0063), whereas metallic containers were associated with normal iodine levels (p=0.0063). Storage time did not significantly influence iodine degradation (p=0.075). There is a deficiency in iodine content of household cooking salt, falling below both the WHO. This finding calls for routine monitoring by relevant authorities and the implementation of preventive measures. Further studies are needed to evaluate the effectiveness of such interventions.

## Introduction

Les troubles dus à la carence en iode (TDCI) sont une série de pathologies regroupant le goitre endémique, l'hypothyroïdie, l'anémie, le retard mental et de croissance chez le nouveau-né et l'enfant, l'hypofertilité, les avortements spontanés et l'accroissement de la mortalité infantile. Par contre, un apport excessif d'iode expose à un risque d'hyperthyroïdie et de maladie auto-immune [1-3]. Bien que l'Organisation mondiale de la Santé (OMS), devant l'ampleur de la carence mondiale avec un tiers de la population carencée [2], a pris une résolution en 1990 pour recommander l'iodation universelle du sel comme stratégie de prévention et de contrôle des TDCI avec comme objectif: plus de 90% de ménages doivent consommés du sel adéquatement iodé (supérieur ou égale à 15 ppm) [3,4]. Cette recommandation a permis une réduction significative du nombre de pays ayant des populations carencées en iode. Cependant, il existe encore ce jour, dans certaines régions du monde présentant une carence d'apport en iode (plusieurs études en ont fait cas dans plusieurs pays). Notamment des régions éloignées de la mer et montagneuses, où sévit le goitre endémique dû à des carences en iode comme celle de certaines régions de la Côte d'Ivoire avec une prévalence globale de 11,3% d'hypothyroïdie [5].

En effet, les zones les plus touchées, qui sont les régions montagneuses du centre-ouest et celles des hauts plateaux du nord, n'ont fait l'objet d'aucune étude d'évaluation du niveau d'iodation au sein de cette population. Par contre, des études d'évaluation ont été réalisées à Abidjan, la capitale économique et ville côtière à la mer, et ont montré que cet objectif de l'OMS reste non atteint [3,6]. Ainsi, Diaby *et al*. (2019) [3] et Adou *et al*. (2002) [6] ont respectivement rapporté 60% et 76,7% de ménages adéquatement iodés, d'où la nécessité de conduire une évaluation régulière de la teneur en iode sur toute la chaîne de distribution (importation, commerce et ménages) et dans certaines populations cibles (femmes enceintes et les enfants). En outre, des auteurs africains, notamment Gomina *et al*. (2011) [2] au Bénin, Banza *et al*. (2016) [1] en RD Congo ont rapporté respectivement 86,24% et 47,5% de sel correctement iodé. Au regard de l'absence d'évaluation en population du niveau d'iodation dans ces régions en proie aux carences en iode et aux TDCI persistants depuis la ratification par la Côte d'Ivoire de l'iodation universelle du sel en 1994 [2,3], nous nous sommes posés des questions à savoir: quelle est la teneur en iode du sel de cuisine, utilisé par la population dans les ménages par rapport aux normes ivoiriennes? Quels sont les facteurs influençant sa consommation et sa conservation? Pour répondre à ces interrogations, une étude auprès des ménages de la ville de Man (chef-lieu de la région de l'ouest) a été réalisée.

## Méthodes

**Cadre et type d'étude:** cette étude a été conduite dans la ville de Man, chef-lieu de la région de l'ouest de la Côte d'Ivoire, située à plus de 500km de la ville d'Abidjan [7]. C'est une étude transversale descriptive qui s'est déroulée sur 1 mois (du 1^er^ au 31 octobre 2023) portant sur le dosage de l'iode dans le sel de cuisine prélevé aléatoirement dans les ménages et dans le commerce avec comme critères principaux de jugement la norme ivoirienne [30 - 50] ppm correspondant à la norme de l'Union Economique et Monétaire Ouest Africaine (UEMOA) et la norme OMS [15 - 30] ppm.

**Echantillonnage:** la taille de notre échantillon a été calculée en utilisant la formule suivante:


$$n=\frac{z^{2}p\left(1-p\right)}{z^{2}}$$

n≈160 ménages inclus: n = taille de l'échantillon, z la constante issue de la loi normale selon un certain seuil de confiance à 95% (z=1,96); p= la proportion de l'hypothyroïdie en Côte d'Ivoire (p=11,3%) [5]; et la marge d'erreur d'échantillonnage choisie (e= 5%). Le critère d'inclusion des ménages était: la disponibilité suffisante de sel de cuisine dans le ménage permettant un prélèvement de la quantité nécessaire et ayant accepté de participer à l'étude après un consentement éclairé. Un prélèvement d'environ 50 g de sel a été effectué dans chacun des ménages inclus dans l'étude de façon aléatoire. Ces prélèvements ont ensuite été conservés dans un pot en polyéthylène stérile de 40ml puis protégés par un sachet en aluminium contre la lumière afin de préserver la teneur en iode. Un questionnaire a été administré par un médecin parlant la langue locale et accompagné d'agents de santé communautaire, dans chaque ménage par interview direct des participants. Tous les échantillons ont été acheminés jusqu'au laboratoire privé du Centre Médical Smires à Abidjan pour être analysés. Le questionnaire comportait les variables suivantes: caractéristiques sociodémographiques, les caractéristiques du sel de cuisine (le lieu d'achat du sel, taille du grain de sel (fin, moyen ou gros), présence ou non d'impuretés dans le sel, la nature de l'emballage de conservation et la durée de conservation).

**Méthode de dosage de l'iode:** le dosage de l'iode par la méthode de titration par iodométrie a été retenue suivant la norme ivoirienne NI: 03 09 003 utilisée dans l'espace de la Communauté Economique des Etats de l'Afrique de l'Ouest (CEDEAO) et UEMOA [3]:

***Principe:*** l'iode contenu dans le sel sous forme d'iodate de potassium est déterminé par la méthode de titration iodométrique. L'iode va réagir avec la solution de thiosulfate de sodium comme suit:


2Na2S2O3+12→2NaI+Na2S4O6

***Légende:*** Na2S2O3: Thiosulfate de sodium; I2: Iode; NaI: Iodure de sodium; Na2S4O6: Tétrathionate de sodium Moléculaire.

***Réactifs:*** solution d'iodure de potassium 10%; acide acétique concentré; thiosulfate de sodium 0,002N; solution d'amidon 1%.

***Mode opératoire:*** peser 10 g de sel préalablement séché et refroidi. Dissoudre 10 g de sel dans 50ml d'eau distillée dans une fiole conique. Ajouter 1ml de solution d'iodure de potassium 10%. Ajouter 1ml de solution d'acide acétique: la solution virera au jaune, cette couleur se développe au fur et à mesure de la libération de l'iode moléculaire. Laisser la réaction se poursuivre pendant environ 10 min à ce qu'elle se termine. Titrée avec la solution de thiosulfate de sodium (0,002N) jusqu'à disparition de la coloration jaune. Ajouter 5ml de solution d'amidon à 1%, une coloration bleue indiquant la présence de l'iode dans la solution. Continuer le titrage jusqu'à disparition de la coloration bleue et noter le volume (V) total utilisé pour le titrage. Expression des résultats: Iode (ppm)= V x 4,2332 où V est le volume de Thiosulfate de sodium 0,002N utilisé pour titrage. Normes CEDEAO- UEMOA: 50 - 80 ppm: importation ou production dans le pays et 30 - 50 ppm: vente en détail et dans les ménages.

**Analyse des données:** les données ont été saisies et analysées par un ordinateur portable muni de logiciels: Epi info dans sa version 7.2.5.0 et Excel pour l'élaboration des figures. Nous avons utilisé comme indicateurs de ces variables: les moyennes pour les variables quantitatives et les proportions pour les variables qualitatives; catégorisé la teneur en iode en intervalle selon les normes ivoiriennes et OMS. Les tests statistiques t-student et Chi-carré ont été utilisés pour comparer les moyennes et les proportions. Les échantillons dont les normes de prélèvement n'ont pas été respectés et les questionnaires incomplets ont été écartés avant la saisie et l'analyse des données.

**Considération éthique:** l'éthique de notre étude est fondée sur la base du respect de la déclaration des principes généraux d'Helsinki de juin 1964. Le protocole a fait l'objet de validation par le comité national d'éthique de Côte d'Ivoire. La participation était conditionnée par un consentement éclairé avec une notice d'information présentant la problématique du sujet et les objectifs à atteindre à l'issue de l'étude. Les données personnelles recueillies n'ont été utilisées que dans le cadre strict de l'étude et protégées dans un micro-ordinateur sous le contrôle de l'investigateur principal.

## Résultats

**Figure 1:**
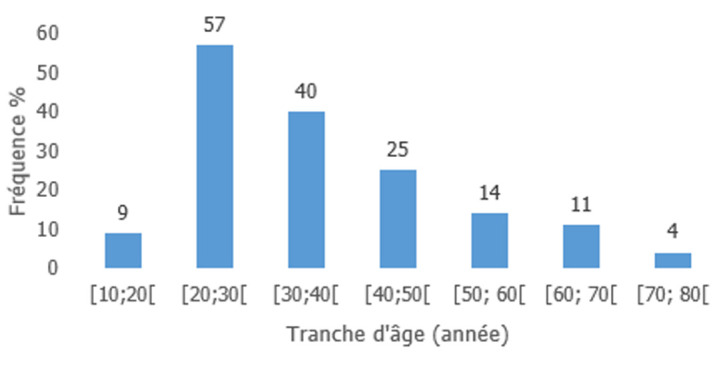
fréquence des tranches d'âge des participants (n=160)

**Caractéristiques sociodémographiques:** l'âge moyen des participants était de 35,6 ans ±13,6 avec un minimum de 16 ans et maximum de 74 ans (n=160). La tranche d'âge de 20 à 30 ans était la plus représentée dans 35% des cas (cf. Figure 1). La majorité, soit 95% des personnes interrogées, était des femmes; la moitié d'entre elles était des ménagères, sans niveau d'instruction ni aucun revenu financier mensuel (Tableau 1).

**Tableau 1:** caractéristiques sociodémographiques des participants dans les ménages

	Effectif (n)	Fréquence (%)	IC à 95%
**Sexe**	**160**		
Féminin	152	95	[90,4 ; 97,8]
Masculin	8	5	[2,2 ; 9,6]
**Résidence**	**160**		
Urbaine	160	100	[97,7 ; 100]
Rural	0	0	-
**Niveau d’instruction**	**160**		
Alphabétisé	13	8,1	[4,4 ; 13,5]
Aucun	75	46,9	[38,9 ; 54,9]
Primaire	35	21,9	[15,7 ; 29,1]
Secondaire	34	21,2	[15,2 ; 28,4]
Supérieure	3	2,00	[0,4 ; 5,4]
**Profession**	**160**		
Chômeur	1	0,6	[0,02 ; 3,4]
Secteur informel	60	37,5	[30 ; 45,5]
Ménagère	79	49,4	[41,4 ; 57,4]
Salarié	12	7,5	[3,9 ; 12,7]
Elève/Etudiant	5	3,1	[1,0 ; 7,1]
Retraité	3	1,9	[0,4 ; 5,4]
**Statut matrimonial**	**160**		
Concubinage	5	3,1	[1,02 ; 7,14]
Célibataire	9	5,6	[2,6 ; 10,4]
Marié	140	87,5	[81,4 ; 92,2]
Veuve	5	3,1	[1,0 ; 7,1]
Divorcée	1	0,6	[0,02 ; 3,4]
**Revenu mensuel**	**160**		
<60 000	58	36,5	[29,0 ; 44,5]
>100 000	10	6,3	[3,1 ; 11,3]
60 000 à 100 000	4	2,5	[0,7% ; 6,3]
Aucun	87	54,7	[46,6 ; 62,6]

### Caractéristique du sel de cuisine

**Moyens de conservation:** nous avons observé une fréquence d'achat du sel de plusieurs fois par semaine dans 31% des cas, puis des achats mensuels dans 21% des cas, avec une quantité variant de 500 à 1000g dans 43,4% suivie d'une quantité de 100 à 200 g dans 27% des cas (n=160). Le sel fin était utilisé pour la cuisine dans 96% des ménages (n=160), avec une absence d'impuretés dans 88% des sels recueillis (n=160). Quant à la conservation du sel au niveau des revendeurs détaillants, elle se faisait à l'aide d'emballages en sachet en polyéthylène (PP) ou en polyéthylène de basse densité (PEBD) scellés dans 100% de cas (n=160). La conservation à domicile se faisant dans la majorité des cas à l'aide de flacons en plastique (65,2%) suivie des sachets PP ou PEBD (26%) avec une durée en moyenne de conservation de 20,5 jours ±23,6 [1; 90 jours] (n=160; voir Tableau 2). Les ménages se procurent du sel auprès des revendeurs détaillants dans 97,5% des cas et chez les commerçants grossistes dans 2,5%.

**Tableau 2:** caractéristiques du sel de cuisine dans les ménages

Caractéristiques du sel de cuisine	Fréquence %	Effectif n=160
**Fréquence achat sel**		
1 fois/mois	21,3	33
> 1 fois par semaine	31	48
1 fois/semaines	19,4	30
1 fois tous les 2 à 3 mois	12,3	19
2 à 3 fois/mois	12,9	20
Une fois tous les 4 à 6 mois	3,23	5
**Quantité de sel acheté (g)**		
< 100	22,6	36
[100 à 200 [	27	43
[200 à 500[	3,8	6
[500 à 1000[	43,4	69
≥ 1000 g	3,14	5
**Taille grain sel**		
Fin	97	152
Gros	1	2
Moyen	2	3
**Propreté du sel**		
Absent d’impureté	88	139
Coloration suspecte (blanc salle noir)	10	15
Corps étrangers (objets)	2	3
**Emballage à l’achat**		
Scellé	99,4	159
Sans scellé	0,6	1
Sachet en polyéthylène	100	160
**Emballage à domicile**		
Scellé	98,8	158
Sans scellé	1,2	2
Boite métallique	1,2	2
Flacon plastique	64,4	103
Flacon verre	7,5	12
Sachet polyéthylène	26,9	43

### Teneur en iode sel iode alimentaire

**Figure 2:**
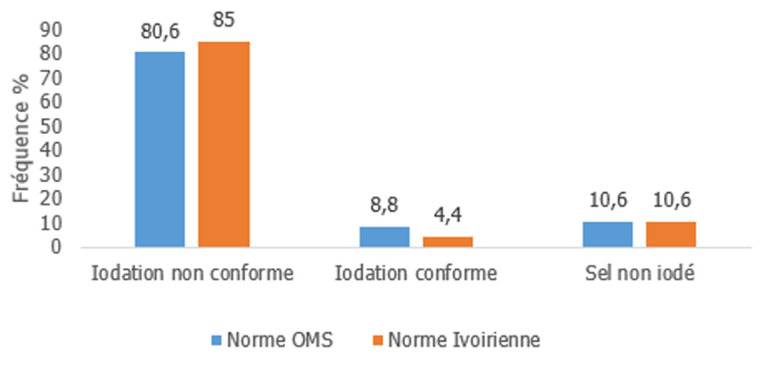
conformité des échantillons selon la norme ivoirienne versus OMS (n=160; p>0,05)

***Au niveau du ménage:*** dans notre série, le dosage de l'iode dans le sel de ménage, objective une teneur en iode en dessous de la norme selon l'OMS (<15 ppm) dans 80,6%, dans la norme (15 à 30ppm) 9% et 11% de sel non iodé. Selon la norme ivoirienne, 85% en dessous (< 30 ppm), 4% dans la norme (30 à 50 ppm) et 11% de sel non iodé dans les ménages (n=160). Aucun cas d'excès d'iodation n'a été observé. Les deux normes ont présenté des résultats similaires, sans différence statistiquement significative, p>0,05 (Figure 2).

***Au niveau des grossistes:*** dans la ville, nous avons constaté la présence de deux grossistes au grand marché. Les sels en cristaux qu'ils importent du Sénégal et de la Guinée sont écrasés en sel fin par des moulins avant de les disponibiliser aux revendeurs détaillants dans les marchés et quartiers. Ce qui explique le fait que la quasi-totalité des sels de ménage est du sel fin. La teneur en iode dans le sel prélevé chez les grossistes est en dessous de 15ppm dans le sel fin et dans le sel en cristaux.

**Figure 3:**
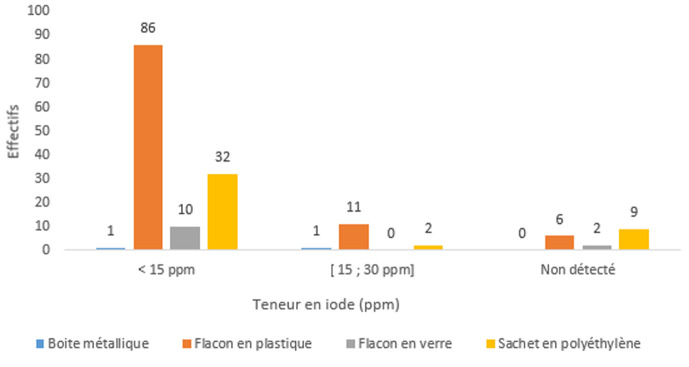
influence du type d'emballage à domicile sur la teneur en iode du sel de cuisine (n=160, p=0,04); ppm=partie par million

**Facteurs influençant la teneur en iode:** les flacons en plastique et en polyéthylène transparents ont un impact significatif sur la teneur du sel de cuisine (p=0,0063). Plus de 80% des sels conservés dans ses flacons ont une teneur en iode en dessous de la norme de l'OMS (Figure 3). Il n'y a pas de lien statistiquement significatif entre la durée de conservation du sel et le niveau de la teneur en iode du sel de cuisine (p=0,075) (Tableau 3).

**Tableau 3:** teneur en iode et durée de conservation du sel de cuisine dans les ménages

	Teneur en iode (ppm)				
Durée conservation (en semaines)	< 15	[15 - 30]	Non détecté	Effectif n=160	P
**<2**	65	3	10	78	0,075
**[2 ; 4 ]**	24	6	1	31	
**[4 ; 8]**	25	1	5	31	
**[8 ; 12]**	8	2	0	10	
**>12**	7	2	1	10	

## Discussion

### Caractéristiques du sel de cuisine

**Moyens de conservation:** Le sel était acheté dans 97,5% chez les revendeurs détaillants ou en boutique et 2,5% chez les grossistes. Aussi, nous avons constaté une utilisation du sel fin pour la cuisine dans 96% des ménages, avec une absence d'impuretés dans 88% des sels recueillis dans les ménages. Puis, la conservation du sel au niveau des revendeurs détaillants se faisait à l'aide d'emballages en sachet en polyéthylène à basse densité ou en polypropylène (PP) avec scellés dans 100% des cas. La conservation à domicile se faisait à l'aide de flacons en plastique (65,2%) suivie des sachets en polyéthylène à basse densité (26%). Le sel y était conservé en moyenne 20,5 jours ±23,6 à l'abri de la lumière dans les ménages. L'iodation du sel dans un pays se doit de respecter la norme de la teneur en iode recommandée par ce pays jusqu'au moment de sa consommation. La stabilité de l'iode dans le sel dépend de plusieurs facteurs à savoir: la substance iodée utilisée, l'emballage, les conditions climatiques environnantes et la période écoulée entre l'iodation et la consommation dans les ménages. Afin de garantir que le sel iodé atteigne les consommateurs avec la concentration d'iode adéquate, des mesures sont mises en place par les pays pour minimiser les pertes importantes d'iode sur toute la chaine de distribution: des emballages en sacs hermétiques en polyéthylène de haute densité (PEHD) ou en polypropylène (PP) ou des sacs de jute doublés de polyéthylène de faible densité sont utilisés; les conditions de stockage et de son transport à respecter. Les sacs qui ont déjà été utilisés pour emballer d'autres substances ne doivent pas être réutilisés pour emballer le sel iodé. Le réseau de distribution est mis en place de sorte à réduire l'intervalle entre l'iodation et la consommation du sel. Le consommateur doit être informé qu'il doit stocker le sel iodé de manière à le protéger d'une exposition directe à l'humidité, à la chaleur et à la lumière du soleil [8], d'où la nécessité de mettre en place un programme de communication et de sensibilisation efficace au sein des populations des zones d'endémie goitreuse dans notre pays.

### Teneur en iode du sel de cuisine

***Au niveau ménage:*** dans notre série, nous avons rapporté que 81% des ménages dans la ville de Man avaient une teneur en iode inférieur à 15 ppm. La norme de la teneur en iode dans le sel exigé par l'OMS est inférieure de 15 ppm [9]. Par contre selon la norme ivoirienne (compris entre 30 ppm et 50 ppm) 85% avait une teneur en iode inférieur à 30 ppm. Certaines études réalisées à Abidjan à savoir celle de Adou *et al*. en 2002 [6] montrant sur 400 ménages à Marcory (Abidjan), 23,3% inférieur à la norme, 44,8% supérieur à la norme et 32% dans la norme. Diaby *et al*. en 2019 [3], révélait auprès de 50 revendeurs détaillants à Marcory (Abidjan), que 40% était en dessous de la norme, 18% de sel conforme et 42% au-dessus de la norme. Nous constations qu'il y'a moins de sel sous dosé dans la région côtière d'Abidjan par rapport au sel consommé dans la ville de Man, qui est une région montagneuse et situé à plus de 500km de la mer. Cette différence entre les deux villes pourrait s'expliqué par le fait qu'Abidjan soit une ville industrielle où l'iodation du sel est réalisée et aussi la dégradation de l'iode du sel par la chaine de distribution (transport sur longue distance) et de conservation (stockage, emballage utilisée). La ville de Man serait donc plus exposée à la dégradation de l'iode, dont les conséquences sont les anomalies liées aux TDCI.

Dans d'autres régions d'Afrique le niveau d'iodation est supérieure au notre mais qui reste toujours en dessous des normes recommandées par l'OMS (qui voudrait que 90% de sel soient dans la norme). Au Bénin Gomina *et al*. en 2011 [2] rapportait 11,31% de sel <15 ppm, 2,45% de sel non iodé, 54,74% conforme à la norme. En RD Congo, Banza *et al*. en 2016 [1] rapportait 47,5% dans la norme, 36,9% faiblement iodés, 7,4% fortement iodé, 8,1% non iodés. En fin au Niger Mamane *et al*. en 2013 rapportait des résultats similaires aux nôtres au niveau ménage à savoir 84,7% de sel dont la teneur en iode est en dessous de 15 ppm; 11,3% répondant à la norme, et 4% au-dessus [10]. Dans d'autres région du monde, des études réalisées au Sri Lanka et en chine respectivement par Kumarasiri *et al*. [11] qui rapportait 31,6% de sel conforme et Wang *et al*. qui rapportait 71,4% de sel conforme [12]. Ces résultats, quand bien même supérieurs aux résultats de notre étude, indiquaient clairement que les recommandations mondiales (OMS) et nationales sur l'iodation du sel n'étaient pas atteintes.

***Au niveau des commerces:*** dans cette ville nous avons constaté la présence de deux grossistes au grand marché, les sels en cristaux qu'ils importent du Sénégal et en Guinée, sont écraser en sel fin à l'aide des moulins avant la distribution aux revendeurs détaillants dans les marchés et quartiers; Ce qui explique le fait que la quasi-totalité des sels de ménages soit du sel fin. La teneur en iode dans le sel prélevé chez les grossistes est en dessous de 15ppm dans le sel fin et dans le sel en cristaux. Dans l'étude menée par Mizéhoun-Adissoda *et al*. en 2018, au Benin, aucun des échantillons de sel chez les grossistes ne respectait les recommandations (20-60 ppm) du sel iodé à la vente pour 190 échantillons de sel prélevés chez les grossistes et revendeuses [13].

**Facteurs influençant la teneur en iode:** dans notre série, les flacons en plastique et les sachets en polyéthylène à basse densité (PEBD) ou en polypropylène (PP) transparents ont eu un impact significatif sur la teneur du sel de cuisine (p=0,0063). Plus de 80% des sels conservés dans leurs flacons ont une teneur en iode en dessous de la norme de l'OMS. Par contre, 50% de sel stocké dans les emballages en boite métallique sont dans la norme et semblerait conservé mieux l'iode que les emballages en flacons plastiques polyéthylène ou polypropylène (PP). Par contre, selon Mahamat *et al*. [14] en 2016, les emballages utilisés pour le sel dans les marchés tels que le polyéthylène à haute densité (PEHD: déperdition 4,60%) et le PP (déperdition 4,48%), sont bien meilleurs et ne sont pas à l'origine de déperditions significatives d'iode si celui-ci est du KIO3. Le polypropylène (PP) à une opacité de densité zéro (donc transparent), a donné la même déperdition que les autres emballages comme le triplex (avec une déperdition à 4,10%; opacité de 2,4) et duplexe (déperdition 4,48%). En outre, Taga *et al*. (2004) [15] au Cameroun rapportait que la bouteille en verre avait une meilleure conservation du sel iodé que le plastique; Gomina *et al*. (2011) confirme également dans leur étude, que la majorité des sels de cuisine entreposés dans les flacons en verre avait une teneur moyenne en iode plus élevée que celle des sels en sachet et en plastique [2]. Enfin, Djonga *et al*. (2012) affirment également que le meilleur récipient est celui fait en verre avec couvercle adapté [9]. Dans notre étude, il n'y a pas eu de lien statistiquement significatif entre la durée de conservation du sel et le niveau de la teneur en iode du sel de cuisine (p=0,075). Dans l'étude de Mahamat *et al*. (2016), le suivi du taux d'iode en fonction de l'emballage au laboratoire durant 12 semaines, le pourcentage de déperdition d'iode était le même pour tous les emballages (triplex, duplex, PEHD et PP) et variait entre 3,98 et 4,73%. La déperdition d'iode était de 3,85% pour le triplex, 4,35% pour le duplex, 3,98% pour le PEHD et 4,85% pour le PP [14].

**Limites de l'étude:** nonobstant les limites connues dans notre étude, nos résultats sont interprétables et ont été discutés avec les données de la littérature. Ces limites sont: d'une part, la présence de deux commerces (grossistes) seulement qui ravitaillent toute la ville, ce qui a pu réduire les écarts de variation de la teneur en iode sur les échantillons prélevés. D'autre part, géographiquement, l'étude a concerné essentiellement les quartiers urbains de la ville Man, et ne pourrait être généralisé de manière précise dans les zones rurales de la commune et en générale de toute de la région Ouest de la Côte d'Ivoire.

## Conclusion

A l'issue de notre étude, l'insuffisance d'iodation du sel de cuisine en dessous des normes de l'OMS et ivoirienne est une réalité dans la ville de Man, nécessitant un contrôle régulier par les autorités compétentes et la mise en place d'actions préventives. Ces actions peuvent être entre autres: l'élaboration d'un programme de lutte contre les TDCI; renforcer le contrôle régulier de la teneur en iode dans les commerces et au niveau des ménages; renforcer la sensibilisation et la communication en vue d'éduquer la population sur l'importance de la consommation du sel iodé. Elaborer et conduire de nouvelles études pour évaluer l'efficacité du programme de lutte qui sera mis en place.

### Etat des connaissances sur le sujet

- La région ouest de la Côte d'Ivoire est connue d'être une zone d'endémie goitreuse due à la carence en iode, dans le sel alimentaire, le sol et les aliments;
- L'Etat de Côte d'Ivoire a adopté des dispositions réglementaires sur l'iodation universelle prôné par l'OMS depuis 1994 ayant permis une régression des TDCI;
- Deux études réalisées à Abidjan en 2019 et 2002 ont respectivement rapporté 60% et 76,7% sur l'évaluation de teneur en iode dans le sel de cuisine des ménages et au niveau des commerces à Marcory, qui montre que le niveau d'iodation reste encore insuffisant par rapport aux normes et objectif de l'OMS (>90% des ménages ayant du sel adéquatement iodé).

### Contribution de notre étude à la connaissance

- Notre étude a décelé une insuffisance d'iodation du sel alimentaire utilisé dans les ménages à plus 80% des échantillons prélevés, permettant d'avoir une photographie du niveau d'iodation dans une zone n'ayant pas été évaluée depuis longtemps, qui est pourtant réputée une zone d'endémie goitreuse;
- Les échantillons provenant des commerces en grossiste ont montré que les sels commercialisés sont inadéquatement iodés;
- Sur la base des données obtenues, l'élaboration et la mise en œuvre d'un programme de surveillance des sels de commerce et une éducation des populations à utiliser du sel adéquatement iodé sont nécessaires pour lutter efficacement contre les TDCI.

## Remerciements

Nous tenons à remercier la Comité National d'Ethique de la république de Côte d'Ivoire (RCI) pour l'approbation du protocole de recherche et le Ministère de la santé et de l'hygiène publique de la RCI pour l'autorisation de la réalisation de cette étude au sein des ménages.
